# Effects of Methylmercury and Theaflavin Digallate on Adipokines in Mature 3T3-L1 Adipocytes

**DOI:** 10.3390/ijms20112755

**Published:** 2019-06-05

**Authors:** Shubhangi Chauhan, Kriya Dunlap, Lawrence K. Duffy

**Affiliations:** 1 Department of Chemistry and Biochemistry, University of Alaska Fairbanks, Fairbanks, AK 99775-6160, USA; chauhanshubhangi3@gmail.com (S.C.); kldunlap@alaska.edu (K.D.); 2 Institute of Arctic Biology, University of Alaska Fairbanks, Fairbanks, AK 99775-7000, USA

**Keywords:** methylmercury, diabetes, adipokines, adiponectin, resistin, lipid peroxidation, theaflavin digallate

## Abstract

Diabetes is a contributor to morbidity across the globe and is often associated with obesity, metabolic syndrome and other inflammatory diseases associated with aging. In addition to genetic and lifestyle factors, environmental factors such as metals and persistent organic pollutants may increase the severity or lower the threshold of these conditions. In cell culture, methylmercury is toxic to adipocytes and may impact adipokine secretions. In this study, we determined the effects of different concentrations of theaflavin digallate on methylmercury exposed 3T3-L1 adipocytes in cell culture. Secretions of resistin, adiponectin and lipid peroxidation product, 4-hydroxynonenal (4-HNE) were monitored using ELISA assays. Cell morphology of methylmercury and theaflavin-3,3′-digallate treated adipocytes was assessed using Lipid (Oil Red O) staining. Exposure to methylmercury increased the levels of resistin and adiponectin as well as 4-HNE when compared to the control cells. Methylmercury treated cells resulted in smaller number of adipocytes and clumped lipid droplets. These results suggest that methylmercury induces reactive oxygen species leading to development of an inflammatory response. Theaflavin-3,3′-digallate reduced the impact of methylmercury by maintaining the adipocytes morphology and secretion patterns of adiponectin, resistin and 4-hydroxynonenal. With this experimental model system other anti-inflammatory and signaling agents could be tested at the biochemical level before eventually leading to studies in animal models.

## 1. Introduction

Methylmercury (MeHg) is one of the forms of mercury species found in the environment. MeHg is a potent neurotoxin which has the ability to accumulate and biomagnify within a diversified ecosystem. Subsequently, bioaccumulation and biomagnification lead to MeHg exposure to human beings [1]. Eighty to ninety percent of organic mercury content found in human beings is from mercury contaminated fish intake [2]. There is a high risk from MeHg exposure to developing fetus among rural fish-eating communities [3]. Due to global transport and changes in the natural processes, Hg levels have found to be increased in the Arctic; this increase is of a major concern at organism level because rural communities in Alaska rely on fish and marine mammals as their staple food [4,5].

Research to determine effects and mechanism of MeHg has focused on induced neurotoxicity [6,7,8,9], but few studies exist which have investigated the detrimental effect of MeHg on the adipose tissue-related cell types [10,11]. To the best of our knowledge, only one study currently exists which has reported the cytotoxic effects of MeHg on 3T3-L1 adipocytes. Vertigan et al. suggested that 0.4 µM (100 ng/mL) of MeHg increases the secretion of Vascular Endothelial Growth Factor (VEGF), a cytokine critical for the process of angiogenesis, during later stages of pre-adipocyte differentiation [10]. Other than this recent MeHg study, other studies from the years 2003 and 2005 have looked into the effect of inorganic mercury species, mercuric chloride (HgCl_2_). In 2003, Barnes et al. reported the inhibiting effects of mercuric chloride on the adipogenesis of pre - adipocytes using 3T3-L1 cells [12]. Later in 2005, they reported that mercuric chloride not only inhibits the differentiation process, but also increases glucose transport into differentiated 3T3-L1 cells [13].

Currently the mechanism of MeHg toxicity is not well understood in adipocytes. What isknown is a chemical property of MeHgidentified as S-mercuration. MeHg has an affinity towards thiol groups, and covalently binds with accessible thiol groups of membrane proteins which may lead to conformational changes in proteins and potential cellular apoptosis [7]. Furthermore, MeHg modified amino acids are sometimes also recognized as methionine molecules by amino acid transporters making it easier for MeHg to cross the membrane barriers [8].

Epidemiological studies indicated that there is a correlation between MeHg exposure and higher incidences of metabolic syndrome related diseases such as type 2 diabetes, chronic inflammation and hypertension [14,15]. In a recent review, we proposed our reactive species hypothesis and suggested that a 3T3-L1 cell model can be developed to determine the effects of MeHg on metabolic syndrome related diseases such as diabetes. For example, adipokines such as adiponectin and resistin and, 4-hydroxynonenal, a product of lipid peroxidation, along with the observation of morphological changes caused by MeHg exposure could be studied in mature 3T3-L1 adipocytes [11].

In cells, MeHg undergoes a series of reactions which may lead to an increase in basal Reactive Oxygen Species (ROS) levels [2]. However, the absolute absence of ROS can also inhibit differentiation process of pre-adipocytes. A basal level of ROS is necessary for normal physiology of adipocytes [16]. The increase in ROS might initiate other sets of chain reactions which can cause an increase in lipid peroxidation [2]. This increased lipid peroxidation can lead to oxidation stress and to initiation of inflammatory diseases. ROS causes oxidative stress and lipid peroxidation which has been suggested as a cause for insulin resistance associated with obesity and type 2 diabetes [17,18].

4-hydroxynonenal (4-HNE), an α, β-unsaturated hydroxyalkenal, is a common product of lipid peroxidation and is used as a biomarker for oxidative stress in cells [19]. MeHg exposure may increase ROS levels which would increase lipid peroxidation resulting in the greater production of 4-HNE [18], and an inflammation of the tissue. 4-HNE is also found to play a role in obesity, the metabolic syndrome, and associated vascular and neurodegenerative disorder [20]. 4-HNE may also be considered as a link for oxidative stress to insulin resistance [21]. Since low levels of MeHg are present in many foods, and is fat soluble, it is reasonable to hypothesize its relationship to adipokines in the exacerbation of inflammation [22]. Additionally, Timar et al. suggested that adipose tissue secreted cytokines are involved in initiation and stimulation of pro-inflammatory status, which contributes to insulin resistance [23].

White Adipose Tissue (WAT) is now considered as a major endocrine organ [24]. WAT is involved in the regulation of metabolic homeostasis via biologically active proteins, adipokines [25]. Adipose tissue and adipokines were reported to be involved in the pathogenesis of type 2 diabetes, coronary artery disease and even arthritis [26,27,28]. Adiponectin and resistin are dependent on the formation of disulfide bonds and, since MeHg also binds to disulfide bonds, therefore they are highly vulnerable; this reaction with MeHg would impact redox potential [25].

Adiponectin is a complex protein mainly secreted from adipose tissue [25]. Adiponectin is found to be actively involved in the metabolic glucose and energy regulation [29,30,31]. It is involved in obesity related diseases such as metabolic syndrome, type 2 diabetes, hypertension and inflammation [32]. Adiponectin also stimulates fatty acid oxidation, decreases plasma triglycerides and increases glucose sensitivity [33]; and, can also act as an anti-inflammatory factor [34]. Lower levels of adiponectin may lead to insulin resistance and type 2 diabetes [35,36,37]. In contrast, higher levels of adiponectin may lead to anorexia nervosa [36]. Over the last decade, higher secretions of adiponectin in immunity-mediated disease such as, Systemic Lupus Erythematosus, Cystic Fibrosis, Inflammatory Bowel Disease, and Rheumatoid Arthritis have been reported [32,38]. Moreover, Apprahamian et al. also reported increased levels of adiponectin in chronic inflammatory diseases [22]. However, the validations and significance of this reported elevation of adiponectin during a state of inflammation is not been yet completely understood.

Resistin, a more recently discovered adipokine [39], is a slightly less studied adipokine with biological properties opposite to adiponectin [29,33]. Resistin is secreted from stromovascular fraction of adipose tissue and blood monocytes [40]. It circulates in human blood as a dimeric protein linked by a disulfide bond [41]. The disulfide bonds in resistin are prime candidates for reduction and are among the most highly exposed disulfides reported for any naturally existing adipokine’s structure [25]. High levels of resistin circulate in diabetic mice models [42]. It is suggested that resistin links obesity to type 2 diabetes [39,43,44]. Additionally, resistin is an inflammatory marker of atherosclerosis in humans [45].

The adiponectin–resistin ratio has been suggested by various researchers to be an indicator of metabolic risk for obesity [29,33,46]. The secretion levels of adipokines, such as adiponectin and resistin, may be associated with the risk of development of hypertension, type 2 diabetes and metabolic syndrome [15,47].

Based on the existing knowledge and ROS central hypothesis, we also tested a naturally occurring anti-oxidative agent, theaflavin-3, 3′-digallate (TF-3), one of the species of theaflavins, on the MeHg exposed mature adipocytes. Our expectation was that TF-3 might negate the adverse effects of MeHg by lowering the increased ROS levels. TF-3 is a naturally occurring polyphenolic component derived from black tea [48]. It has also been reported to have anti-oxidative, anti-bacterial, anti-inflammatory, anti-angiogenic and anti-cancer properties [49,50]. Moreover, TF-3 was also reported to reduce the incidences of cardiovascular diseases [50]. The remedial effect of TF-3 makes it a promising therapeutic agent for inflammation and cancer related disease, but the mechanism of its specific action has not yet been completely understood.

Since it has been reported that most Alaskan Natives have elevated mercury levels (0.4 µM) [51], and this concentration also affected the VEGF secretion levels [10], we exposed 3T3-L1 adipocytes with a constant volume of MeHg (0.4 µM) to mimic the effects of elevated blood mercury level. For the investigation of TF-3 on adipokine levels and the morphology of 3T3-L1 adipocytes, we selected different TF-3 concentrations as an anti-oxidant [50].

The aim of the research was to explore the primary effects of MeHg and TF-3 on the secretion levels of adipokines. Lipid peroxidation product, 4-HNE at the biochemical level, was used to monitor oxidative stress related to inflammation. The following hypotheses were tested using outcome measures of resistin, adiponectin and 4-HNE levels in 3T3-L1 cultures.

1. MeHg exposure interferes with the normal morphology of mature adipocytes.
2. MeHg exposure to the mature adipocytes will change the normal secretion patterns of adiponectin and resistin.
3. TF-3 in a concentration dependent manner will restore the secretion patterns of adiponectin and resistin secreted from MeHg exposed adipocytes.
4. TF-3 concentrations will reduce the increased level of 4-HNE secreted from MeHg exposed mature adipocytes in a concentration dependent manner.

## 2. Results

### 2.1. Morphological Changes in 3T3-L1 Adipocytes upon Exposure to MeHg and TF-3 Using Oil Red O Staining

**Figure 1:**
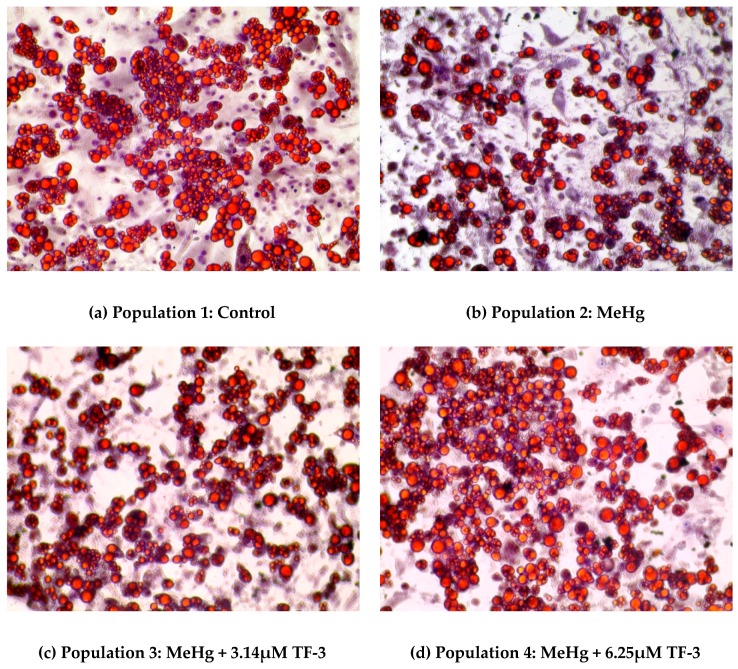
Morphology changes caused due to different treatments to mature adipocytes on Day 28 using lipid staining. (**a**) shows the stained lipids in the control group (Population 1); (**b**) shows the stained lipids under MeHg exposure (Population 2); (**c**) shows the stained lipids exposed to MeHg and 3.14µM TF-3 (Population 3); (**d**) shows the stained lipids exposed to MeHg and 6.25 µM TF-3 (Population 4).

In Figure 1, a set of images of Oil Red O stained 3T3-L1 adipocytes show a change in cell morphology after MeHg and MeHg + TF-3 exposure. Lipid staining helps to differentiate between differentiated and non-differentiated cells. Lipid droplets are indicative of successful differentiation and will turn red in color, while nuclei appear blue in color. Cells in the control population show that red color is dominant suggesting successful maintenance of differentiated 3T3-L1 adipocytes, even at Day 28. The mature cells have spherically distinct lipid droplets. In MeHg exposed adipocytes population, red stained cells are less suggesting smaller number of adipocytes, clumped with smaller lipid droplets. 0.4 µM (100 ng/mL) MeHg altered the normal spherical morphology of the healthy cells resulting in fewer numbers and distortion of adipocytes. Overall from Day 18 to Day 28, MeHg induced apoptosis was evident in population 2 which suggests that MeHg stressed the cells by altering reactive oxygen species as indicated by the presence of 4-HNE. Most of the fat globules had smaller sized droplets and cells were highly agglomerated. Lipid staining on Day 28 of the culture study showed less stained lipid droplets in population 2 (0.4 µM MeHg treatment) when compared to control cells.

In a 3.14 µM TF-3 treated adipocytes population; lesser red stained cells can be seen when compared to the control population. It shows more red stained adipocytes than the MeHg exposed population, suggesting positive effects from TF-3. Increased red stained cells among the 6.25 µM TF-3 treated group can be seen, suggesting TF-3 helped cells to maintain their standard morphology even after exposure to MeHg. Adipocytes in higher TF-3 dosages were found to be similar to the adipocytes in populations 3 and 4; but their cell numbers were slightly less.

Treatment with a range of different concentrations of TF-3 was carried out on MeHg exposed cells. During initial days of treatment (Days 18–24), TF-3 counteracted MeHg induced changes as seen in population 2. Cells at these concentrations of TF-3 populations looked comparatively healthier with less clumping and bigger fat globules than MeHg treated cells. During the last four days (Days 24–28) of study, 6.25 µM TF-3 concentration had the highest number of stained cells when compared to control. Other tea concentrations such as 3.14 µM, 12.5 µM, 25 µM, 50 µM and 100 µM had larger and less clumped lipid droplets but were fewer in number.

### 2.2. Effects of MeHg and TF-3 on the Secretion Patterns of Adiponectin

The mean concentration of adiponectin secretion in control cells (population 1) from Day 18 to Day 28 was 35.8 ± 3.0 ng/mL.MeHg exposure on the mature cells resulted in significant increase in adiponectin levels. The mean adiponectin concentration from Day 18 to Day 28 was 54.1 ± 4.0 ng/mL (*p* < 0.0001, as compared to the control). MeHg + 3.14 µM TF-3 exposure on the mature cells showed significant increase in adiponectin levels. Although it was expected that tea treatment would return the increased adipokine level to control level, 3.14 µM TF-3 increased adiponectin secretion levels even more. The mean adiponectin concentration from Day 20 to Day 28 in MeHg + 3.14 µM TF-3 population was 56.9 ± 6.0 ng/mL (*p* < 0.0001, as compared to the control).

Treatment of MeHg exposed cells with 6.25 µM TF-3 did counteract the MeHg secretion effect, adiponectin level was lower than population 2 (MeHg exposed) because the concentration of adiponectin secretion in population 2 was 54.1 ± 4ng/mL but in population 4 (MeHg + 6.25 µM), it was 50.6 ± 8 ng/mL (*p* < 0.0001, as compared to the control).

Subsequently, in the population 5 group, where the concentration of TF-3 was increased to 12.5 µM, no such noticeable change was seen when compared to treatment with 6.25 µM. The mean adiponectin concentration in population 5 (MeHg + 12.5 µM TF-3) from Day 18 to Day 28 was 51.6 ± 4.2 ng/mL (*p* < 0.0001, as compared to the control).

Adiponectin levels in population 6 (MeHg + 25 µM TF-3) were found to be closest to the control cells’ adiponectin levels. MeHg exposure, which caused a significant increase in adiponectin secretion levels seen in population 2, was partly restored by treatment of cells with 25 µM TF-3. The collective mean concentration of adiponectin calculated in population 6 from Day 18 to Day 28 was 41.4 ± 5.0 ng/mL (*p* = 0.0006, as compared to the control). In populations 7 and 8, as the adiponectin levels continued to decrease. Adiponectin concentrations for populations 7 and 8 were calculated as 13.4 ± 15 ng/mL (*p* = 0.0023, as compared to the control) and 8.9 ± 9.0 ng/mL (*p* < 0.0001, as compared to the control) respectively.

**Figure 2:**
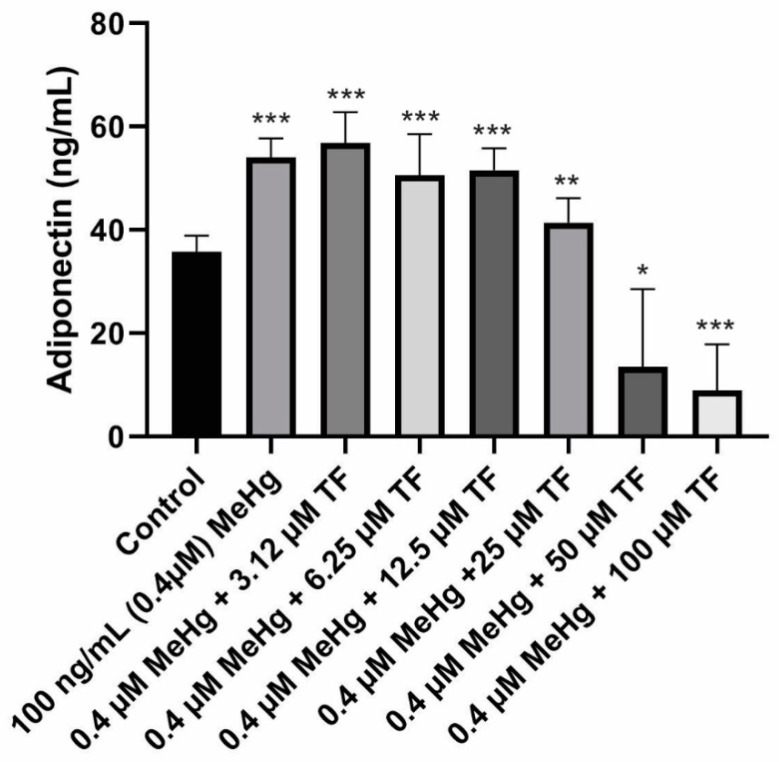
Adiponectin secretion in all eight populations from Day 18 to Day 28. Overall statistically significant difference was determined using the Kruskal–Wallis method and *p*-value was less than 0.0001. Differences are considered significant (*) if p-value is less than or equal to 0.00625, very significant (**) if less than or equal to 0.001 and extremely significant (***) if less than or equal to 0.0001.

To determine the overall statistical significance of data obtained from various populations, Kruskal–Wallis analysis of variance was performed. The reported *p*-value was less than 0.0001 and the entire study was found to be extremely and significantly different. Adiponectin concentration in both concentration ends of the treatment with TF-3 resulted in either higher value or lower values as compared to the control. Treatment of MeHg exposed cells with 25 µM TF-3 turned out to be the most appropriate and efficient concentration. The 25 µM restored the adiponectin concentration closer to the control group. Other TF-3 concentrations such as 3.14, 6.25 and 12.5 µM comparatively increased secretion levels, while 50 and 100 µM TF-3 concentrations greatly decreased adiponectin levels (Figure 2).

### 2.3. Effects of MeHg and TF-3 on the Secretion Patterns of Resistin

The mean concentration of resistin secretion in control cells (population 1) from Day 18 to Day 28 was calculated as 8781.4 ± 705.0 pg/mL. In comparison, the adiponectin level from the control cell population was 35.8 ± 3.0 ng/mL (~35,000 pg/mL). Smaller quantities of resistin are secreted as compared to adiponectin secretion from standard mature 3T3-L1 adipocytes under similar in vitro conditions.MeHg exposure on the mature cells caused an increase in resistin levels during the exposure study. The mean resistin concentration from Day 18 to Day 28 was calculated as 9717.3 ± 3914.0 pg/mL (*p* = 0.3453 as compared to the control).

A large decrease in the resistin release was seen during the last four days of the culture study. Due to this sudden fall, the standard deviation within each population increased which prevented the increase from being significant at *p* ≤ 0.05. This variation was observed in all the eight different cell populations.

In contrast to the 3.14 µM TF-3 effect on adiponectin secretion, resistin secretion at 3.14 µM TF-3 was similar to the control group’s resistin secretion.The mean resistin concentration in population 3 (MeHg + 3.14 µM TF-3) from Day 18 - Day 28 was calculated as 8841.5 ± 5349.0 pg/mL (*p* = 0.4864, as compared to the control). Similar concentrations seen in populations 1 and 3suggest that the sensitivity of resistin to a small amount of TF-3, effectively restoring the resistin level after MeHg exposure.

Since 3.14 µM TF-3 had restored the resistin levels tobeing close to the control cells, it was expected that this higher concentration of TF-3 would further decrease resistin levels. It turned out that it did decrease the resistin concentration even more, resulting with resistin secretion smaller than the control group. The mean resistin concentration in population 4 (MeHg + 6.25 µM TF-3) from Day 18 to Day 28 was calculated as 7616.0 ± 4489.0 pg/mL (*p* = 0.5125, as compared to the control). The TF-3 reduced the level of increased resistin concentration, counteracting some of the effects of MeHg. While the resistin concentration in population 3 suggested a positive aspect of treatment with TF-3, any greater concentration of TF-3 than 3.14 µM dropsresistin levels lower than the control group. The mean resistin concentration in population 5 (MeHg + 12.5 µM TF-3) from Day 18 to Day 28 was 5964.6 ± 4387.0 pg/mL (*p* = 0.3669, as compared to the control). Downward secretion patterns of resistin werefurther seen in higher TF-3 doses (25, 50 and 100 µM).

**Figure 3:**
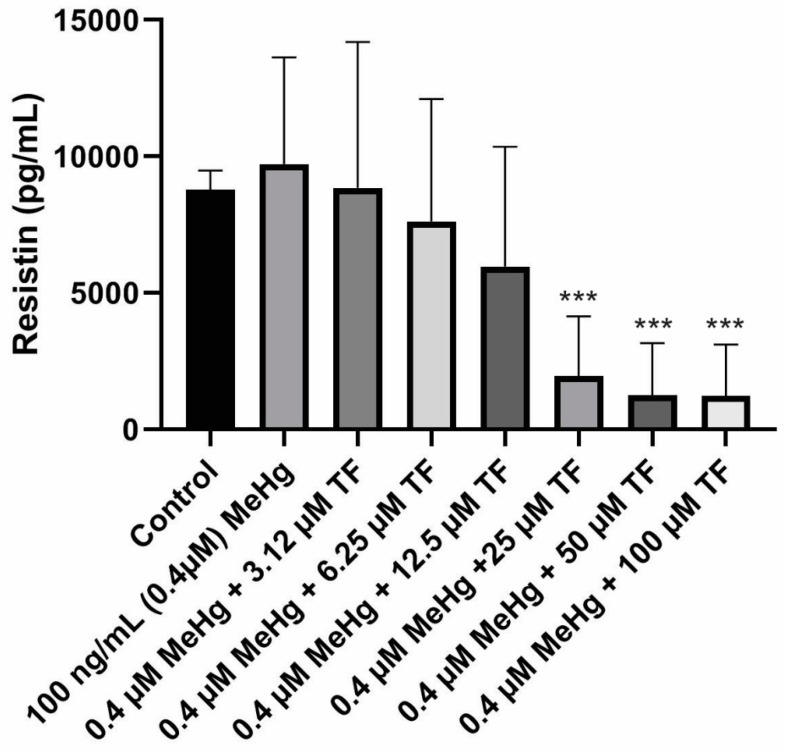
Resistin secretion in all eight populations from Day 18 to Day 28. Overall statistically significant difference was determined using the Kruskal–Wallis method and *p*-value was less than 0.0001. “***” shows an extremely significant difference having a *p*-value less than or equal to 0.0001.

In the case of adiponectin, only 50 and 100 µM TF-3 decreased the adiponectin to levels lower than the control group. The 6.25 µM TF-3 reduced the secretion level to lower than the control group. The mean resistin concentration in population 6 (MeHg + 25 µM TF-3) from Day 18 to Day 28 was 1956.2 ± 2183.0 pg/mL (*p* < 0.0001, as compared to the control). The effects of 50 and 100 µM TF-3 on MeHg induced change in adipokine secretions further. The resistin concentration in population 7 (MeHg + 50 µM TF-3) from Day 20–Day 28 was 1255.0 ± 1899.0 pg/mL (*p* < 0.0001, as compared to the control). The mean resistin concentration in population 8 (MeHg + 100 µM TF-3) from Day 18 to Day 28 was 1224.4 ± 1882.0 pg/mL (*p* < 0.0001, as compared to the control). Resistin’s concentration ranged between 8781.4 ± 705.0 pg/mL and 1224.4 ± 1882.0 pg/mL (Figure 3).

The combined effects of MeHg and TF-3 (at higher concentrations) had a negative effect on signaling. The variation in the scale seen in the secretion patterns for resistin is complex. A declining pattern was seen but the reduced resistin levels below the control level would lead to an imbalance in normal signals.

### 2.4. Effects of MeHg and TF-3 on the Secretion Patterns of 4—Hydroxynonenal

Basal levels of ROS are vital for the differentiation and maintenance of adipocytic cells, ROS production triggers the rate of lipid peroxidation, thereby causing production of 4-HNE. 4-HNE concentration in the control 3T3-L1 adipocyte population over the course of ten days was 4.4 ± 1.1 ng/mL. Treatment of mature adipocytes with 0.4 µM MeHg resulted in increased 4-HNE production (11.90 ± 2.1 ng/mL; *p* = 0.0012 as compared to the control). The increased 4-HNE production suggests an increase in lipid peroxidation activity induced by MeHg. It could also be related to increase in reactive species production. As hypothesized, treatments with different concentrations of TF-3 were found to be effective at reducing the increased 4-HNE production induced by MeHg.

Treatment of 3.14 µM TF-3 on MeHg treated cells resulted in almost similar levels of 4-HNE production as reported in the control group. A 2.2 ± 2.2 ng/mL 4-HNE was produced from 3.14 µM TF-3 treated population (*p* = 0.1370, compared to the control). Treatment of 6.25 µM TF-3 also turned out to be effective because it resulted in lower production of 4-HNE as compared to MeHg treated population. The mean 4-HNE concentration in 6.25 µM TF-3 treated cellular population was 7.2 ± 2.0 ng/mL (*p* = 0.0006, as compared to the control). These findings suggest that TF-3 has the potential to counteract MeHg induced oxidative stress at the cellular level. Data for treatments with 12.5 and 25 µM TF-3 have not been included because of the higher standard deviation (more than 2 standard deviation from the mean value) among the 4-HNE data points.

**Figure 4:**
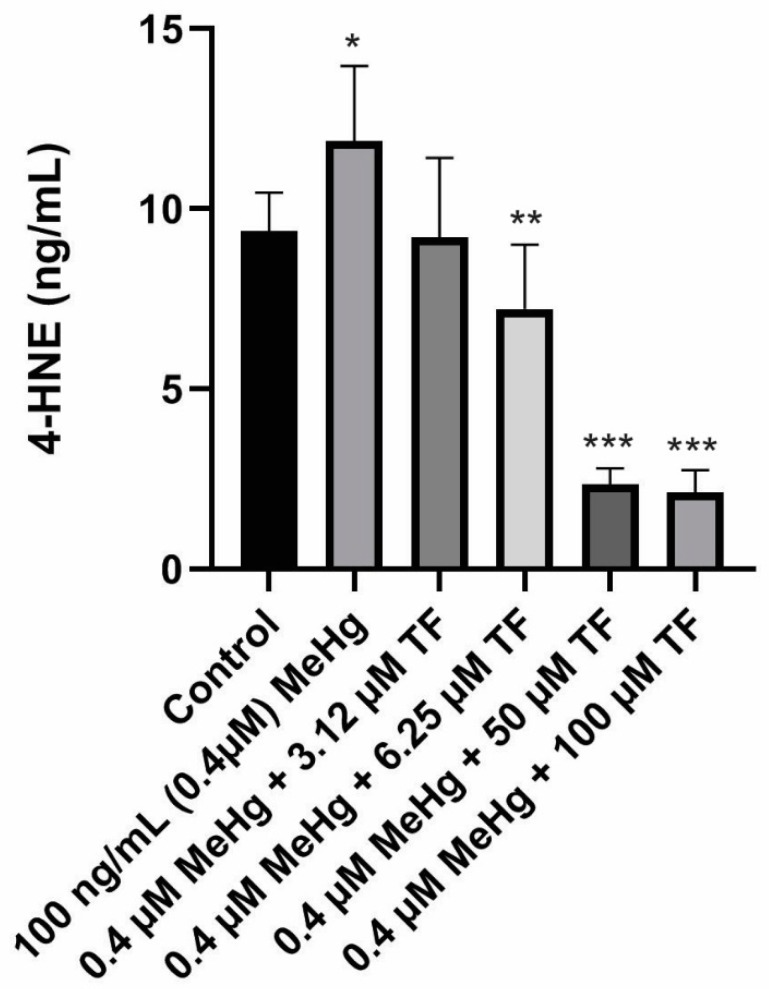
4-HNE secretion in all six populations from Day 18 to Day 28. Overall statistically significant difference was determined using the Kruskal–Wallis method and *p*-value was less than 0.0001. Differences are considered significant (*) if p-value is less than or equal to 0.00625, very significant (**) if less than or equal to 0.001 and extremely significant (***) if less than or equal to 0.0001.

Treatments with higher TF-3 doses 50 and 100 µM produced 4-HNE lower than that of the control group. The mean 4-HNE in population MeHg + 50 µM TF-3 was 2.3 ± 0.5 ng/mL (*p* < 0.0001 as compared to the control). Treatment of adipocytes with 100 µM TF-3 did not produce a difference in 4-HNE concentration from 50 µM TF-3 treated cells. The mean 4-HNE levels in the highest TF-3 concentration was 2.1 ± 0.6 ng/mL (*p* < 0.0001, as compared to the control), which was lower than the control and MeHg treated populations. From these findings, it supports that TF-3 be considered a potential anti-inflammatory agent for treating MeHg induced oxidative stress (Figure 4).

## 3. Discussion

Increasing rates of type 2 diabetes and hypertension are often associated with obesity with mild and/or chronic inflammatory conditions [2,18]. We addressed the question of whether MeHg promotes the pro-inflammatory signs leading to a chronic inflammatory disease state. An inflammatory condition is reported in metabolic syndrome and cardiovascular diseases [14,32]. Since adipokines commonly play roles in these diseases and are considered as biomarkers for inflammation, oxidative stress and metabolic syndromes, the effect of an anti-inflammatory natural product to counteract 4-HNE and return the adipokines to control levels was tested. Additionally, three lower concentrations of TF-3 (3.14 µM, 6.25 µM and 12.5 µM) were added to the control group from Day 18 to Day 28. When the collected medium was analyzed for the quantification of adiponectin and resistin, it was found that the secretions levels were similar to that of the quantities of adiponectin and resistin secreted from the control group. The effects of higher concentrations of TF-3 on the adipokines secretions were not tested.

This study determined the effects of MeHg and TF-3 on the morphology, the secretions of two adipokines and 4-HNE levels in 3T3-L1 adipocytes. MeHg initiated an increase in ROS production, which eventually increases lipid peroxidation and the generation of 4-HNE, a stable product of lipid peroxidation [2,18]. The TF-3 concentrations showed a dose dependent response in maintaining normal cell morphology in the MeHg exposed cells. TF-3 neutralized some of the increased 4-HNE level and reduced the elevated oxidative stress. Further analyses of the effect of TF-3 on MeHg exposed cells will give a clearer picture. An increase in ROS levels is a possible explanation leading to an increase in 4-HNE, a lipid peroxidation product.

Adiponectin is one of the biomarkers reported to determine normal metabolic status of the human body [29]. Adiponectin levels are associated with metabolic syndrome related diseases, cardiovascular diseases and autoimmune diseases [22,36]. In this study, MeHg exposure increased levels of adiponectin when compared to control cells. This increase suggested an increase in inflammatory activities induced in a cell system, since adiponectin can act as an anti-inflammatory and anti-oxidative [37,52]. Earlier reports suggest that adiponectin increased in chronic inflammatory related diseases [22,32,38]. Our results suggest that higher adiponectin levels are associated with an increase in inflammatory and oxidative conditions in exposed cells.

In the MeHg treated cells, some TF-3 concentrations showed their anti-oxidative properties by reducing the increased concentration of adiponectin while 3.14 µM TF-3 did not counteract the effects of 0.4 µM MeHg. The 6.25 µM and 12.5 µM TF-3 were able to reduce the increased adiponectin levels. The best response was obtained from 25 µM TF-3 since it restored the increased adiponectin levels close to that of the control cells. The 50 and 100 µM TF-3 decreased adiponectin levels to even lower than that of the control cells suggesting greater concentrations of TF-3 are not an appropriate dosage in combatting the MeHg exposure. High concentration of TF-3 might interfere with the natural ROS signals.

Compared to adiponectin, resistin is a more recently discovered adipokine. It is still under study to explore its role in cellular processes. It may be a link that connects obesity and type 2 diabetes [39,43]. Resistin is an inflammatory marker for cardiovascular diseases [29,45]. Higher levels of resistin are found in a condition of insulin resistance [42]. Our data showed that MeHg increased the mean resistin levels in exposed 3T3-L1 cells. Resistin levels were drastically decreased during the last four days (Days 24–28), but overall there was an increase in resistin concentration. This suggests that cells were under oxidative stress. Since resistin is an inflammatory factor, the higher resistin levels were seen in MeHg treated adipocytes population. The fact thatresistin levels decreased during the last four days, but thatadiponectin levels remained increased in a gradual way, might indicate that an autocrine feedback mechanism is present.

Resistin levels were very sensitive to TF-3 treatment because even 3.14 µM TF-3 was effective in restoring mean resistin levels close to the control cells’ resistin level. Higher TF-3 concentrations decrease resistin levels gradually. Further research will provide more reasoning behind this effectiveness. The 25, 50 and 100 µM TF-3 reduced resistin levels drastically lower than the control cells. The best TF-3 concentration which restored the increased resistin level was 3.14 µM. This concentration neutralized the increased ROS levels in MeHg treated cells, thereby reducing oxidative stress.

A recent study by Ko et al., reported TF-3 induced augmentation of AMPK activity. The enhanced AMPK activity leads to decrease in free fatty acid release. More importantly, increased AMPK activity inhibits NFkB activity and also down-regulates pro-inflammatory cytokines such as TNF-α and IL-6. Altogether, changes in the activity of AMPK caused by TF-3 can hinder the NFkB chronic inflammation cascade [53].

4-HNE is a product of lipid peroxidation and increases in cells under oxidative stress [18,19]. Increased levels of 4-HNE have been reported in aging, diabetes, obesity and neurodegenerative diseases [54]. Our results support the concept of 4-HNE as an oxidative stress marker and its increase in this study supports that adiponectin and resistin are active in the redox balance. MeHg exposed cells showed fewer numbers of adipocytes than the control group, which suggests that MeHg exposure did not cause obesity among the cells.

In obesity, when type 2 diabetes occurs, resistin levels increase and adiptonectin decreases. In cell culture exposed to MeHg, resistin also increased suggesting an inflammatory response. The lipid staining demonstrated clumping of adipocytes but no hypertropicadipoctytes. Adiponectin levels increased similar to that reported in obesity. Ko et al., (2014) also reported that TF3 enhanced the response of adiponectin in co-cultured adipocytes. TF3 reduced both resistin and adiponectin in the MeHg cultures.

Determination of particular reactive oxygen and nitrogen species such as hydrogen peroxide, superoxide, will be more beneficial for determining exact difference before and after exposure. This could further be supported by testing the response of anti-oxidants levels such as catalase, superoxide dismutase to the exposure of MeHg. These experiments would support our central hypothesis that MeHg increases basal ROS levels, which further causes lipid peroxidation and affects various interconnected cellular processes. Cell morphology could also be assessed by using different analytical methods; methods such as measuring optical densities of the stained lipids in MeHg exposed and control groups. Further specific analysis could be performed using flow cytometry method analyzing physical and chemical characteristics of MeHg treated adipocytes. Furthermore, determining the effect of MeHg on the adipocytes morphology can also be achieved by measuring mature adipocytes expressed markers such as fatty acid binding protein 4 (FABP4).

## 4. Materials and Methods

A common cell type used for in vitro studies for the phenomenon of adipogenesis and adipokines is the 3T3-L1 cell line, a well-characterized adipocyte line from mice which can acquire an adipocyte-like phenotype. During the differentiation process, 3T3-L1 cells can be induced to differentiate, under controlled laboratory conditions, using a standard differentiation protocol; and, the cell line is widely accepted as a physiologically faithful in vitro representation of adipogenesis [10,13,55].

### 4.1. Cell Culture

3T3-L1 pre-adipocytes were purchased from American Type Culture Collection (ATCC). The thawed vial from ATCC was subcultured using the standardized chemical induced differentiation protocol (Chemically-Induced Differentiation of ATCC^®^ CL-173™ (3T3-L1), 2011). After 72 h, cells were counted using a hemocytometer (Weber Scientific, Hamilton Township, NJ, USA). Equal number of cells (approximately 35,000 cells per 2 mL liquid medium per well), were plated in Falcon 12-well flat-bottom cell culture plates (Thermo Fisher, Santa Clara, CA, USA). Optimal conditions for humidified incubation of the cells were 5% carbon dioxide along with a constant a temperature of 37 °C.

**Figure 5:**
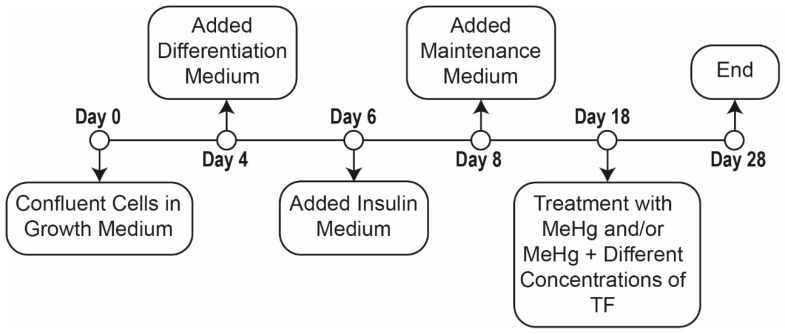
Timeline of the exposure study.

Triplicates of eight distinct cell populations were established (Table 1). Populations were comprised of: (1) control, (2) MeHg treated and (3) MeHg + six different concentrations of theaflavindigallate (TF-3) treatments. Initially, cells were grown to 80–90% confluence in growth medium for 48 h; confluent cells were then incubated for another 48 h in the growth media. Subsequently, the cells were incubated in differentiation media for 48 h, followed by the incubation for another 48 h in insulin medium. Finally, all eight populations were incubated in maintenance medium for the next 10 days to obtain mature adipocytes. Before replacing or switching medium, 1.5 mL of the liquid media was collected in 2 mL eppendorf tubes and stored at −20 °C until analysis (Figure 5).

The concentration of MeHg used for the treatment was 100 ng/mL (0.4 µM), as we aimed to mimic the elevated blood mercury levels found in Alaskan Native populations [51]. Vertigan et al. reported that 100 ng/mL MeHg altered VEGF secretion levels in 3T3-L1 cells [10].

Theaflavin-3, 3′-digallate (TF-3) is a reported anti-oxidant and anti-inflammatory molecule. The effect of TF-3 on cancerous cells has been reported using different concentrations [50]. Similar TF-3 concentrations were used in this study to measure the effect of TF-3 on MeHg exposed 3T3-L1 adipocytes (Table 1).

### 4.2. Treatment with Toxin and Antioxidant

Treatment with MeHg and TF-3 was performed for a course of ten days after cells matured (Figure 5, Days 18 to 28). The control cells were neither treated with MeHg nor any concentration of tea compound, TF-3, for the entire 28-day course of cell culture. MeHg treatment on 3T3-L1 adipocytes population was performed from Day 18 through to Day 28 (population 2). Different concentrations of TF-3 (3.14, 6.25, 12.5, 25, 50 and 100 µM) were also tested on 0.4 µM MeHg exposed cells from Day 18 through to Day 28 (Table 2).

MeHg stock solution was purchased from Alfa Aesar having a concentration of 1000 ppm in Milli-Q water. For obtaining the final concentration of MeHg, initial dilution to 1 ppm (1000 ng/mL) was performed by adding 1.4 µL of MeHg from stock bottle in 1.4 mL of prepared maintenance medium. To obtain the final MeHg working concentration to 100 ng/mL, 200 µL of the diluted MeHg solution (1000 ng/mL) was added to each well having 1.8 mL of the maintenance medium or maintenance medium plus appropriate volumes of tea compound. Fresh solutions having MeHg and/or tea compound were made each day shortly before addition to wells. The leftover and unused solutions were discarded properly as hazardous waste.

TF-3 was purchased from LKT LABS. On arrival, the tea compound, a brick red colored powder was used to make a stock solution of 1 ppm (1000 ng/mL) in dimethyl sulfoxide (DMSO, Sigma Aldrich, St. Louis, MO, USA). Appropriate volumes of stock solution were used to obtain desired concentrations required for the treatment in each mentioned population. Table 3 lists the volume specifications for MeHg and TF-3 along with medium used and maintained in each well. The total liquid volume comprising MeHg and/or TF-3 along with maintenance medium was maintained at 2 mL. Table 3 outlines the composition of populations 1 to 8 in culture well plates.

An EVOS XL Core Imaging System, a transmitted light microscope, was used to view cells and capture their images. Cells were imaged before changing and replacing culture medium. Control cells were imaged from Day 0 to Day 28 and treated cells were imaged from Day 18 to Day 28. “Day 2” images represent the appearance of the cells after 48 h of incubation with Growth Medium; “Day 4” images show cells after 48 h in Differentiation Medium (Figure 5).

### 4.3. Safety

Appropriate personal protective equipment was used at all times while working with MeHg solutions. Silver Shield gloves, along with an additional outer nitrile glove, were worn whenever concentrated MeHg stocks were opened, as well as disposable lab coats and a chemical safety hood. All mercury-containing liquid or solid wastes, including cell media, Falcon tubes, Eppendorf tubes, and pipet tips, were disposed of as hazardous waste according to local regulations.

### 4.4. Morphology:Lipid (Oil Red O) Staining

A lipid staining kit was used for selective staining and detection of lipid droplets in matured 3T3-L1 cells (matured adipocytes). Oil red O is a lysochromediazo dye used for staining lipids. Hematoxylinwas included in the kit stains the nuclei of the cells. The Lipid (Oil Red O) Staining Kit contained PBS, 10% formalin, Oil Red O and hematoxylin (Sigma Alrich). Other reagents and supplies required were Whatmann No. 1 filter paper, 60% isopropanol and 100% isopropanol. The experimental protocol is briefly described below:

On Day 28 of cell culture, immediately after the media collection from all of the wells, cells were ready for staining. The staining assay started with the remaining cell culture media in the wells being removed. After removal of cell culture media, cells were washed twice with PBS. After the wash, 10% formalin was pipetted down on to the sides of wells. The formalin treated cells were incubated for 45 min. After the incubation period, formalin was discarded and cells were washed twice with water. 60% isopropanol was added to wells and incubated for 5 min. After discarding the 60% isopropanol, cells were evenly covered with Oil Red O working solution (was prepared 15 min before use by adding, mixing and filtering 3 parts of Oil Red O stock solution to 2 parts of water). The well plate was gently rotated and incubated for 15 min. After the incubation period, the Oil Red O solution was discarded and stained cells were washed five times with water until no excess stain was seen. After obtaining clear cells, hematoxylin was added to the cells and incubated for 1 min, after which thehematoxylin was discarded and the cells were washed five times with water. Cells were covered with water and observed under a microscope. Images were captured at the time of observation. Lipid droplets appeared red and nuclei appeared blue.

### 4.5. Enzyme Linked Immunosorbent Assay (ELISA)

The Enzyme Linked Immunosorbent Assay (ELISA) is a highly specific and sensitive technique used for quantitatively determining the analyte or protein of interest in a given biological sample. It works on the principle of specific binding of an antigen to its antibody. There are different types of ELISA kits available in the market based on the type of antigen-antibody binding; the most commonly used principle is “sandwich ELISA”. Another type also used in this study is “competitive ELISA”. The analytes of interest in this study were adiponectin, resistin and, 4-Hydroxynonenal (4-HNE). Commercially available ELISA kits were purchased from R&D Systems, and MyBioSource, respectively.

#### 4.5.1. Sandwich ELISA: Adiponectin and Resistin

The R&D kit provides its own protocol, which is briefly described below:

The frozen cell media from different populations were thawed at room temperature, after which, the thawed eppendorf tubes containing cell media were centrifuged at 2000 rpm for five minutes to remove particulates. The kit provided a known protein standard which was serially diluted to generate a standard curve. The possible adiponectin detection range was 0 ng/mL–10 ng/mL; resistin detection range was 0 pg/mL–200 pg/mL. The samples were diluted to 1:3 with calibrator diluent before loading them into ELISA microwell plate. The purpose of sample dilution was to obtain absorbance readings within the known standard curve. A total of 50 µL of standards, blank or diluted samples were added to the protein antibody coated microplate, followed by microplate incubation for 3 h at room temperature, after which the microplatewas rinsed five times using a wash buffer. Later, 100 µL of conjugate antibody was added to the microplate and the plate was incubated for another one hour at room temperature. The microplate was again washed five times with wash buffer after which 100 µL of the substrate solution was added to the plate and incubated for another 30 minin the dark. The last step of the process was the addition of the stop solution which generated a yellow color; the absorbance was read at 450 nm in a BioTek Synergy HT plate reader using Gen5 software.

#### 4.5.2. Competitive ELISA: Mouse 4-Hydroxynonenal (4-HNE)

The MyBioSource kit provides its own protocol, which is described briefly below.

Frozen cell media from different population were thawed at room temperature, after which, the thawed eppendorf tubes containing cell media were centrifuged at 2000 rpm for five minutes to remove particulates. A kit provided a known 4-HNE reference standard which was serially diluted and used to generate a standard curve. The possible 4-HNE detection range was 0 ng/mL–10 ng/mL. The samples were diluted to 1:2 with sample diluent before loading them into ELISA microwell plate. The complete experiment was performed at 37 °C. A total of 50 µL of standards, blank or diluted samples were added to the 4-HNE antigen coated well plate. Immediately, 50 μL of biotinylated detection antibody was added to the well pate which was later sealed and incubated for 45 min at 37 °C. Afterwards, the microplatewas rinsed three times using a wash buffer. A total of 100 µL of horse radish peroxide (HRP) conjugate solution was added to the microplate and the plate was incubated for another 30 min at 37 °C. The microplate was again rinsed five times with wash buffer after which 90 µL of the substrate solution was added to the plate and incubated for approximately 15 min in the dark. Similar to the adiponectin and resistin ELISAs, the last step of the process included the addition of the stop solution terminating the ongoing enzyme substrate reaction; the absorbance was read at 450 nm in a BioTek Synergy HT plate reader using Gen5 software

### 4.6. Statistical Analysis

The cell culture setup included triplicates of each population (population 1 to 8). Graphpad Prism, version 8.0.1 software was used to prepare all the graphs and to perform statistical analyses. For the analysis of adipokines and 4-HNE secreted from the treated populations (population 1 to 8) cell culture media was collected from Day 20 to Day 28. The samples were collected at a regular interval of 48 h. The cells’ maintenance medium during Days 18–28 were used to compare the adiponectin secretion between treated and non-treated cell populations. The concentration levels were calculated based on the standard curve obtained from respective ELISA assays.

The normal secretion patterns of adipokines and 4-HNE is represented by bar graphs, comprising values of mean ± standard deviation obtained from three different experiments. Each day (from Day 20 to Day 28) has three values or data points, which are averaged to obtain mean values and to calculate standard deviation.

To determine the statistically significant difference between the secretion levels of adiponectin, resistin and 4-HNE from the non-treated and treated cell populations, overall effect is considered instead of the effect on a single day. Three replicates from each day (Day 20, Day 22, Day 24, Day 26 and Day 28) are analyzed collectively, each population had a total of 15 data points (*n* = 15), comprising values obtained from three different experiments.

To determine the normality of the data, the Shapiro–Wilk test was used. Normally distributed data is analyzed using parametric tests. To determine the overall statistical significance, one way analysis of variance (ANOVA) is applied. Values are found to be statistically significant different if the *p*-value is less than 0.05.

To determine the significant difference between two populations, (e.g., control cells vs. MeHg treated cells or control cells vs. MeHg+ 3.14 µM TF-3 etc), Student’s T test is used, and data are found to be significant if the *p*-value is less than 0.05. In order to correct for multiple comparisons, Bonferroni corrections is performed on all *T*-tests.

Non-normal data are analyzed using non parametric methods. To determine the overall statistically significance, Kruskal–Wallis one-way analysis of variance is applied. Values are found to be statistically significant if the *p*-value is less than 0.05. However, to determine the significant difference between two populations, (e.g., control cells vs. MeHg treated cells or control cells vs. MeHg+ 3.14 µM TF-3 etc), a Mann–Whitney U test was used and data are noted to be significant if *p*-value is less than 0.05. Similarly, in order to correct multiple comparisons, Bonferroni corrections is performed on all pairs of columns.

Data are presented as mean ± standard deviation unless indicated otherwise. Alpha value is taken as 0.00625 instead of 0.05 after Bonferroni correction. Differences are considered significant (*) if *p*-value is less than or equal to 0.00625, very significant (**) if less than or equal to 0.001 and extremely significant (***) if less than or equal to 0.0001.

## 5. Conclusions

A cell system model to determine the effects of MeHg and TF-3 on adipocytes was developed. This model can be used to determine the effects of other heavy metals on adipokines secretion levels. The mechanism of gene activation by MeHg or any other heavy metal should be explored. Future studies should also include working on the unknown mechanism of MeHg toxicity in different cell types. Different adipokines other than adiponectin and resistin can be studied to investigate MeHg induced changes. Different TF-3 concentrations can be used to have a clearer understanding of the anti-oxidative effects of polyphenolic compounds. Determining effects of different polyphenols extracted from natural sources in withstanding detrimental effects of heavy metals on cell types will provide a promising nutraceutical or therapeutic research pathway for treatment.

## Figures and Tables

**Table 1:** List showing study groups of different populations.

Population (All Populations are Plated as Triplicates)	Treatment *
Population 1	Control
Population 2	100 ng/mL (0.4µM) MeHg
Population 3	0.4 μMMeHg + 3.12 µM TF-3
Population 4	0.4 μMMeHg + 6.25 µM TF-3
Population 5	0.4 μMMeHg + 12.5 µM TF-3
Population 6	0.4 μMMeHg + 25 µM TF-3
Population 7	0.4 μMMeHg + 50 µM TF-3
Population 8	0.4 μMMeHg + 100 µM TF-3

* Note: For populations 2 to 8, treatment started on Day 18 to Day 28. Media from Day 18 to Day 28 were stored and eventually analyzed for resistin, adiponectin and 4-HNE (Figure 5).

**Table 2:** Cell Culture Protocol.

Day Name	Procedure/Medium Used
	Grow cells to confluence in Growth Medium; replace mediumevery 2–3 days.
Day 0	Cells are confluent. Add Growth Medium; incubate 48 h.
Day 2	Change Growth Medium; incubate 48 h.
Day 4	Add Differentiation Medium; incubate 48 h.
Day 6	Add Insulin Medium; incubate 48 h
Day 8	Add Maintenance Medium; incubate and change every 48 h for 8–10 days until mature adipocytes are formed.
Day 18–Day 28	Add Maintenance medium, methylmercury (100 ng/mL) and range of concentrations of theaflavin-3, 3′-digigallate (3.14 µM, 6.25 µM, 12.5 µM, 25 µM, 50 µM and 100 µM); incubate and change every 48 hours for the next 10 days.

**Table 3:** Liquid media composition of culture well plates.

Control	2000 µL Growth Medium
0.4 µM MeHg	1800 µL growth medium + 200 µL MeHg
MeHg+ 3.14 µM TF-3	1794.58 µL growth medium + 200 µL MeHg+ 5.42 µL TF-3
MeHg+ 6.25 µM TF-3	1789.14 µL growth medium + 200µL MeHg+ 10.86 µL TF-3
MeHg+ 12.5 µM TF-3	1778 µL growth medium + 200 µL MeHg+ 22 µL TF-3
MeHg + 25 µM TF-3	1756.56 µL growth medium + 200 µL MeHg+ 43.44 µL TF-3
MeHg + 50 µM TF-3	1713 µL growth medium + 200 µL MeHg+ 87 µL TF-3
MeHg +100 µM TF-3	1626 µL growth medium + 200 µL MeHg+ 174 µL TF-3
